# Establishment and Characterization of a Chicken Myoblast Cell Line

**DOI:** 10.3390/ijms25158340

**Published:** 2024-07-30

**Authors:** Dongxue Guo, Shudai Lin, Xiaotong Wang, Zhenhai Jiao, Guo Li, Lilong An, Zihao Zhang, Li Zhang

**Affiliations:** 1 College of Coastal Agricultural Sciences, Guangdong Ocean University, Zhanjiang 524088, China; 2 Key Laboratory of Farm Animal Genetic Resources and Germplasm Innovation in Zhanjiang, Guangdong Ocean University, Zhanjiang 524088, China

**Keywords:** chicken, myoblast cell line, *chTERT*, G418 screening, proliferation and differentiation

## Abstract

Skeletal muscle, which is predominantly constituted by multinucleated muscle fibers, plays a pivotal role in sustaining bodily movements and energy metabolism. Myoblasts, which serve as precursor cells for differentiation and fusion into muscle fibers, are of critical importance in the exploration of the functional genes associated with embryonic muscle development. However, the in vitro proliferation of primary myoblasts is inherently constrained. In this study, we achieved a significant breakthrough by successfully establishing a chicken myoblast cell line through the introduction of the exogenous chicken telomerase reverse transcriptase (*chTERT*) gene, followed by rigorous G418-mediated pressure screening. This newly developed cell line, which was designated as *chTERT*-myoblasts, closely resembled primary myoblasts in terms of morphology and exhibited remarkable stability in culture for at least 20 generations of population doublings without undergoing malignant transformation. In addition, we conducted an exhaustive analysis that encompassed cellular proliferation, differentiation, and transfection characteristics. Our findings revealed that the *chTERT*-myoblasts had the ability to proliferate, differentiate, and transfect after multiple rounds of population doublings. This achievement not only furnished a valuable source of homogeneous avian cell material for investigating embryonic muscle development, but also provided valuable insights and methodologies for establishing primary cell lines.

## 1. Introduction

The poultry industry has garnered escalating significance within the global economic landscape, including chicken, duck, and goose, which are the major sources of protein in the supplement of meat and egg for people [1]. Additionally, skeletal muscle constitutes an indispensable component of an animal’s body. The proliferation and differentiation of myoblasts to produce multinucleated myofibers, as well as their fusion with existing myofibers, is crucial for skeletal muscle development [2,3]. Given that myogenesis in myoblasts encompasses an intricate, multistage process orchestrated by numerous myogenic regulatory factors [4], it is a long-term research project to reveal the molecular mechanisms underneath to improve the skeletal muscle growth and meat production performance of poultry. Therefore, the high availability of primary myoblasts would make a great contribution to the understanding of the molecular mechanisms in myogenesis. However, the inherent limitation of the proliferation ability of primary myoblasts in vitro poses a challenge to scientific progress in this field. Currently, several skeletal myoblast cell lines, such as rat L6 [5], mouse C2C12 [6], canine Myok9 [7], and grass carp CIM [8], have been successfully established and well used. Nevertheless, the establishment of a poultry skeletal myoblast cell line remains unreported, which underscores the need for the development of a poultry skeletal myoblast cell line. Chicken serves as an ideal model organism for myogenesis research [9]. Therefore, in this study, we aimed to establish a chicken myoblast cell line, which would significantly advance research in the realm of muscle development and production efficiency in the poultry industry.

A prior study claimed that normal animal cells possess a finite lifespan, with limited proliferation capabilities, both in vivo and in vitro, and they would be in a static state after a certain stage, which is a phenomenon known as the Hayflick limit [10]. Subsequent investigations unveiled the role of telomeres, which are the protective structures at the ends of eukaryotic linear chromosomes, in modulating cellular immortality. Telomeres are ribonucleoprotein complexes composed of highly conserved non-coding DNA sequences that feature repetitive guanine-rich base sequences (5′-TTAGGG-3′)_n_ in tandem repeats. These structures are closely associated with a specific set of proteins known as “shelterin” [11]. The distinctive free (5′-TTAGGG-3′)_n_ overhanging sequence at the 3′ end of telomeric DNA undergoes insertion into the double-stranded DNA, forming a lasso-like structure known as the telomere loop (t-loop). This unique structure effectively conceals the chromosome end, providing a shield against recognition as DNA damage [12]. With the progression of research, it has become widely believed that the “shelterin” protein complex comprises six key components: telomere repeat binding factors 1 and 2 (TRF1, TRF2), protection of telomeres 1 (POT1), tripeptidyl peptidase 1 (TPP1), TRF1 interaction nuclear factor 2 (TIN2), and repressor/activator protein 1 (RAP1). These six proteins engage in intricate interactions with telomeric DNA, either directly or indirectly, to enhance the stability of the t-loop structure. This fortification plays a crucial role in protecting the chromosome ends from being recognized as DNA damage signals, thereby maintaining genomic stability [13,14].

Studies conducted in the 1990s unveiled progressive telomere shortening as a fundamental molecular mechanism contributing to cellular aging [12,15]. As cells undergo continued divisions, the “shelterin” protein complex gradually disengages from the shortening telomere. Consequently, the stability of the t-loop structure weakens, leaving the exposed chromosome ends susceptible to recognition as DNA double-strand break damage. This, in turn, activates the p53–p21 pathway and the p16–Rb pathway, leading to cell senescence [12,15]. Currently, it is widely accepted that two distinct mechanisms govern telomere maintenance mechanisms within cells: the telomerase-dependent pathway and the alternative lengthening of telomeres pathway (ALT) [16]. Telomerase, which is a ribonucleoprotein with reverse transcriptase activity, is composed of several components, including telomerase RNA component (TERC), telomerase-associated protein (TEP), and telomerase reverse transcriptase (TERT). Notably, TERC and TERT are indispensable core elements that underpin telomerase functionality. They catalyze the addition of (5′-TTAGGG-3′)_n_ repeat sequences to the 3′-OH end of DNA, facilitating the synthesis of new telomeric DNA, which is crucial for maintaining the integrity of linear chromosome ends [17]. Research indicated the widespread presence of TERC and TEP in most human tissues and cells. In contrast, the *TERT* gene exhibits tissue-specific expression, primarily in tissues and cells characterized by robust self-renewal capabilities, including stem cells and germ cells [18]. *TERT* expression is generally considered a hallmark of telomerase activation. It has been reported that the expression of the *hTERT* in normal human myoblasts was sufficient to yield telomerase activity to elongate telomeres, but it failed to produce immortalization [19]. Generally, chicken cells can hardly immortalize spontaneously because of their low spontaneous mutation rate [20]. Telomeres play an important role in maintaining chromosome stability and determining the cellular life span [21]. And the introduction of the human telomerase reverse transcriptase (*hTERT*) gene into cells, as well as the restoration of telomerase activity, represent an ideal method for cell immortalization [22]. Furthermore, a previous study established two immortalized chicken preadipocyte cell lines by the retroviral transduction of *chTERT* and *chTERC* [23]. Therefore, in the present study, we embarked on a pioneering endeavor by successfully establishing a chicken myoblast cell line through the introduction of the heterologous expression of the chicken telomerase reverse transcriptase (*chTERT*) gene into the cells. This groundbreaking achievement introduced a valuable source of homogeneous cell material for studying embryonic muscle development in non-mammalian vertebrate model organisms.

## 2. Results

### 2.1. Isolation of Chicken Primary Myoblasts and Determination of G418 Optimal Concentration

**Figure 1:**
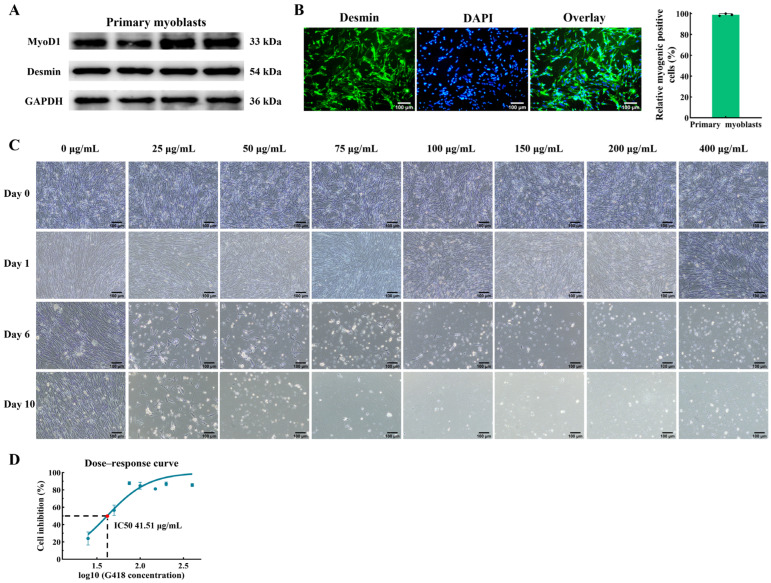
Myogenic identification of chicken primary myoblasts and cell screening by G418 with different concentrations. (**A**) Relative protein expression levels of MyoD1 and Desmin in the primary myoblasts after inducing differentiation for 48 h. (**B**) Primary myoblasts were stained with the myogenic cell marker Desmin (green) and DAPI (blue). The scale bar represents 100 μm. (**C**) The chicken primary myoblasts were screened by different concentrations of G418. “Day 0” represents the cells after 24 h of proliferation, and “Day n (n ≠ 0)” represents the cells after n days of G418 screening. Magnification 100×, scale bar 100 μm. (**D**) The determination of the minimum lethal dose of G418 on the chicken primary myoblasts.

First, we isolated primary myoblasts from thigh muscle and used them for the myoblast cell line establishment. In order to identify the cell type, the expression of key myogenic proteins (MyoD1 and Desmin) in the primary myoblasts after inducing differentiation were quantified by Western blot. As a result, the expressions of MyoD1 (myogenic orientation determining factor) and Desmin (marker protein specifically expressed by myogenic cells) were found to be positive in the primary myoblasts (Figure 1A). In addition, immunofluorescence analyses demonstrated that the purity of the myogenic cells obtained through the differential adhesion method exceeded 90% (Figure 1B), thereby validating the suitability of the isolated primary myoblast population for subsequent cellular screening procedures.

Generally, studies use antibiotics to screen the cell lines that stably express the target gene [24,25,26]. To establish the chicken myoblast cell line, we used the antibiotic G418 to screen the cells. There are differences in the tolerance of different cells to G418. In order to ensure that only the cells with the inserted gene survived after transfection, the G418 concentration gradient was first set up for pre-experiment to determine the optimal lethal concentration of G418 on chicken primary myoblasts. In this study, the optimal screening concentrations of G418 for chicken primary myoblasts were determined through the cell morphology and CCK-8 assay. The morphological analysis showed that no inhibition of cell growth was observed on the first day under the concentrations of G418 in the chicken primary myoblasts of 25, 50, 75, 100, 150, 200, and 400 μg/mL (Figure 1C). However, after 6 days of screening, only a few cells survived at these concentrations, and all chicken primary myoblasts died by the 10th day of G418 screening (Figure 1C). Upon exposure to a G418 concentration of 25 μg/mL, the primary myoblasts could survive after 10 days of screening (Figure 1C). In addition, the OD450 values of the cells before administration and 6 days after the addition of G418 were measured, and the proliferation inhibition rates of chicken primary myoblasts were calculated for different concentrations of G418. A dose–response curve was generated to assess the concentration of G418 that caused a 50% reduction in the chicken primary myoblasts proliferation (Figure 1D). As a result, the optimal concentration for the G418 screening in the chicken primary myoblasts was determined to be 41.51 μg/mL (Figure 1D).

### 2.2. Establishment of Chicken Myoblast Cell Line

**Figure 2:**
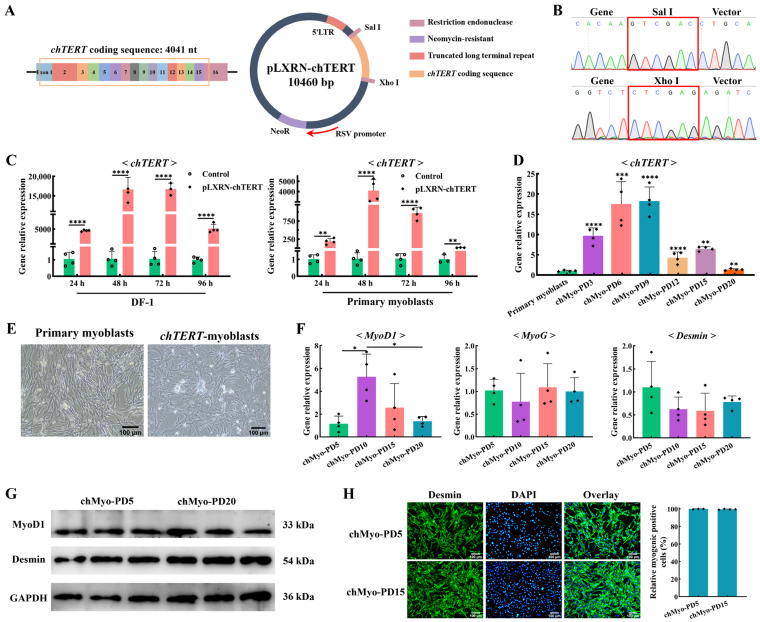
Immortalization of chicken primary myoblasts and cell line identification. (**A**) Structure diagram of the pLXRN-chTERT plasmid. (**B**) Sequencing results of the pLXRN-chTERT plasmid. The red box indicates different restriction site. (**C**) The relative expression levels of *chTERT* in different chicken-derived cells transfected with the pLXRN-chTERT for 24, 48, 72, and 96 h (mean ± SD; ** *p* < 0.01; **** *p* < 0.0001; *n* = 4). (**D**) The relative expression levels of *chTERT* in chicken primary myoblasts and *chTERT*-myoblasts at different population doublings (chMyo-PDs) (mean ± SD; ** *p* < 0.01; *** *p* < 0.001; **** *p* < 0.0001; *n* = 4). (**E**) Light microscopy of chicken primary myoblasts (**left**) and *chTERT*-myoblasts (**right**). (**F**) The relative expression levels of *MyoD*, *MyoG*, and *Desmin* in *chTERT*-myoblasts at different PDs after inducing differentiation for 48 h (mean ± SD; * *p* < 0.05; *n* = 4). (**G**) Relative protein expression level of MyoD1 and Desmin in *chTERT*-myoblasts at PDs 5 and 20 after inducing differentiation for 48 h. (**H**) *chTERT*-myoblasts at PDs 5 and 20 were stained with the myogenic cell marker Desmin (green) and DAPI (blue).

We overexpressed the *chTERT* gene in chicken primary myoblasts using the pLXRN-chTERT retroviral expression vector (Figure 2A). The sequencing results of the vector showed that the *chTERT* coding region (4041 bp) was successfully cloned into the pLXRN vector through the Sal I-Xho I restriction site (Figure 2B). After overexpressing *chTERT* in the chicken DF-1 cells and primary myoblasts with the pLXRN-*chTERT* vector for different durations (24, 48, 72, and 96 h), the relative expression levels of *chTERT* were significantly higher than those in the control group (*p* < 0.01) (Figure 2C), indicating the integrity of the *chTERT* insertion on the pLXRN-chTERT vector and the successful overexpression of *chTERT*.

In order to establish the chicken myoblast cell line, we used G418 selection medium at the optimal concentration (41.51 μg/mL) to screen the cells. After the retroviral infection and G418 selection, the resulting cells were continuously subcultured at a 1:2 split ratio. Chicken myoblasts with the overexpression of *chTERT* proliferated similarly to the primary myoblasts and showed growth inhibition in the first few population doublings. Following the screening process, a small group of the selected cells gained a proliferative advantage and underwent continuous expansion. This cell line that stably expressed *chTERT* was continuously passaged at a 1:2 split ratio and exhibited cumulative population doublings that exceeded 20 (Figure 2D), which far exceeded the maximum in vitro life span of chicken primary myoblasts (population doublings for 2 to 4) [27]. According to the definition of immortal cell lines [28], this cell line could be considered as immortal, and we designated the selected chicken myoblasts that stably expressed *chTERT* as *chTERT*-myoblasts.

No obvious cellular morphological differences were observed between the chicken primary myoblasts and the *chTERT*-myoblasts (Figure 2E), which underscored the fact that *chTERT*-myoblasts maintained the inherent morphological features of chicken primary myoblasts. The myogenic regulatory factors *MyoG* and myogenic marker *Desmin* were positively expressed in the *chTERT*-myoblasts at population doublings (PDs) 5, 10, 15, and 20 with no significant difference (Figure 2F). Correspondingly, the protein expression levels of MyoG and Desmin in the *chTERT*-myoblasts of PDs 5 and 20 were also positively expressed with no significant difference observed (Figure 2G), implying that the cells between different generations maintained stable and similar capabilities for myogenesis and differentiation. It is worth noting that the expression levels of *MyoD1* between *chTERT*-myoblasts at different population doublings were different (Figure 2F), suggesting that our *chTERT*-myoblasts at different population doublings had distinct differentiation rates (differentiation degree for the same differentiation time). With several generations of population doublings, the morphological features between the *chTERT*-myoblasts at PDs 5 and 15 remained consistent, where the purity of myogenic cells in these two cells were approximately 100% (Figure 2H). Furthermore, the expressions of Desmin in the *chTERT*-myoblasts at PDs 5 to 15 were sustained at high levels (Figure 2H), indicating that the *chTERT*-myoblasts exhibited a pure population of myogenic cells throughout the population doublings of this cell line.

### 2.3. chTERT-Myoblasts Retained the Proliferation Capacity of Chicken Primary Myoblasts

**Figure 3:**
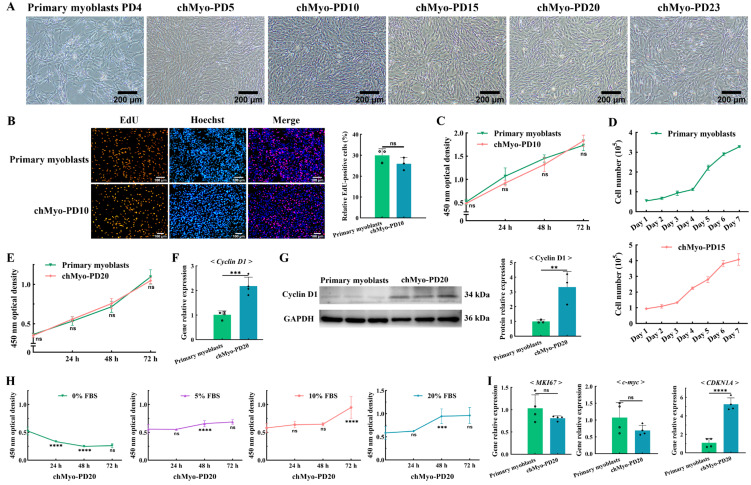
Proliferation characteristics of *chTERT*-myoblasts. (**A**) Light microscopy of chicken primary myoblasts and *chTERT*-myoblasts at different population doublings (chMyo-PDs). (**B**) Proliferation of chicken primary myoblasts and *chTERT*-myoblasts at PD 10 were assessed by EdU (mean ± SD; ns represents non-significant; *n* = 3). (**C**) CCK-8 assay on chicken primary myoblasts and *chTERT*-myoblasts at PD 10 (mean ± SD; ns represents non-significant; *n* = 6). (**D**) Cell growth curve of chicken primary myoblasts and *chTERT*-myoblasts at PD 15 (mean ± SD; *n* = 3). (**E**) CCK-8 assay on chicken primary myoblasts and *chTERT*-myoblasts at PD 20 (mean ± SD; ns represents non-significant; *n* = 6). (**F**) Relative expression level of proliferation marker gene in *chTERT*-myoblasts at PD 20 by qPCR (mean ± SD; *** *p* < 0.001; *n* = 4). (**G**) Relative protein expression level of proliferation marker gene in *chTERT*-myoblasts at PD 20 (mean ± SD; ** *p* < 0.01; *n* = 3). (**H**) Serum-dependent analysis on *chTERT*-myoblasts at different population doublings (mean ± SD; ns represents non-significant; *** *p* < 0.001; **** *p* < 0.0001; *n* = 6). (**I**) Relative expression level of oncogenes and tumor suppressor genes in *chTERT*-myoblasts at PD 20 by qPCR (mean ± SD; ns represents non-significant; **** *p* < 0.0001; *n* = 4).

With the establishment of the chicken *chTERT*-myoblast cell line, we further assessed its proliferation characteristics. It was known that the chicken primary myoblasts could only be continuously subcultured for PDs 2 to 4, while the *chTERT*-myoblasts were successfully subcultured and maintained the normal morphology of myoblasts to at least PD 23 (Figure 3A). The proliferation assay revealed that there was a higher number of EdU(+) cells in chicken primary myoblasts compared with the *chTERT*-myoblast at PD 10 (*p* > 0.05) (Figure 3B), indicating that the chicken primary myoblasts proliferated faster than the *chTERT*-myoblasts at PD 10. Furthermore, the CCK-8 assay also demonstrated that the growth kinetics of chicken primary myoblasts was faster than that of the *chTERT*-myoblasts at PD 10 (*p* > 0.05) (Figure 3C). However, cell-counting analysis revealed that the growth curve trajectory of the *chTERT*-myoblasts at PD 15 was analogous to that of chicken primary myoblasts, and *chTERT*-myoblasts exhibited an earlier onset of the rapid proliferation phase compared with the primary myoblasts (Figure 3D). Similarly, the CCK-8 assay demonstrated that the proliferation profile of *chTERT*-myoblasts at PD 20 was comparable with that of the chicken primary myoblasts, and with a higher proliferation rate compared with the primary muscle cells during the early growth phase (Figure 3E). Furthermore, the expression levels of the myoblast proliferation marker gene *Cyclin D1* was significantly higher in the *chTERT*-myoblasts at PD 20 compared with the primary myoblasts after proliferation for 24 h (Figure 3E). Correspondingly, the protein expression levels of Cyclin D1 was significantly higher in the *chTERT*-myoblasts at PD 20 (Figure 3F). These findings collectively suggest that while the *chTERT*-myoblasts maintained a proliferation profile analogous to that of the primary myoblasts, their proliferation rate differed from the primary cells with different population doublings.

It is widely known that normal somatic cells cultured in vitro rely on serum for their nutritional needs. To delve into the potential cancerization trend of *chTERT*-myoblasts during population doublings, we systematically assessed the cell viability under various serum concentrations conditions. The result indicated that the *chTERT*-myoblasts at PD 20 was unable to grow normally when cultured in 0% FBS (Figure 3G). With the FBS concentration at 5% or 10%, the *chTERT*-myoblasts at PD 20 exhibited a restoration of normal proliferative capacity and showed an optimal proliferation when cultured in a medium with FBS concentration at 20% (Figure 3H). Furthermore, the expression levels of the oncogenes (*MKI67* and *c-myc*) in the *chTERT*-myoblasts at PD 20 was lower than the primary myoblasts (*p* > 0.05), while the expression levels of the tumor suppressor gene *CDKN1A* in the *chTERT*-myoblasts at PD 20 was significantly higher than the primary myoblasts (Figure 3I). In conclusion, these *chTERT*-myoblasts showed no cancerization phenomenon.

### 2.4. chTERT-Myoblasts Retain the Differentiation Capacity of Chicken Primary Myoblasts

**Figure 4:**
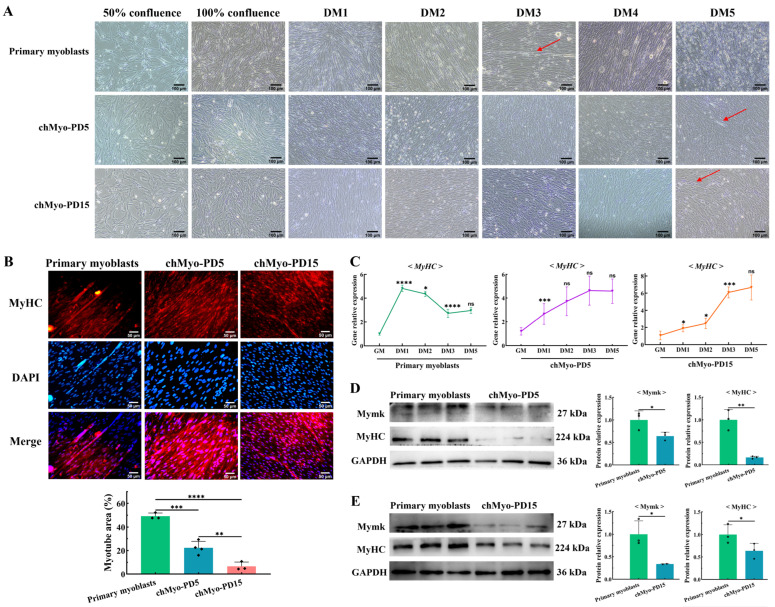
Differentiation characteristics of *chTERT*-myoblasts. (**A**) Light microscopy of chicken primary myoblasts and *chTERT*-myoblasts at PDs 5 (chMyo-PD5) and 15 (chMyo-PD15) in differentiation medium (DM) for different days. The red arrows indicate the formed myotubes. (**B**) After inducing differentiation for 48 h, myotubes derived from chicken primary myoblasts and *chTERT*-myoblasts at PDs 5 and 15 by performing immunofluorescence staining against MyHC (red), and nuclei were counterstained with DAPI (mean ± SD; ** *p* < 0.01; *** *p* < 0.001; **** *p* < 0.0001; *n* = 3). (**C**) Relative expression levels of differentiation marker gene on chicken primary myoblasts and *chTERT*-myoblasts at PDs 5 and 15 in differentiation medium (DM) for different days (mean ± SD; * *p* < 0.05; *** *p* < 0.001; **** *p* < 0.0001; ns represents non-significant; *n* = 4). (**D**) Relative protein expression levels of differentiation marker genes in chicken primary myoblasts and *chTERT*-myoblasts at PD 5 after inducing differentiation for 48 h (mean ± SD; * *p* < 0.05; ** *p* < 0.01; *n* = 3). (**E**) Relative protein expression levels of differentiation marker genes in chicken primary myoblasts and *chTERT*-myoblasts at PD 15 after inducing differentiation for 48 h (mean ± SD; * *p* < 0.05; *n* = 3).

Subsequently, we embarked on investigating the persistence of differentiation potential in *chTERT*-myoblasts across population doublings. Using the differentiation medium (DM) with 2% house serum, these cells were induced to differentiate into myotubes. The light microscopy results demonstrate that similar to the differentiation of chicken primary myoblasts, the *chTERT*-myoblasts at PDs 5 and 15 fused together to form nascent myotubes (Figure 4A). Comparatively, the myotubes were observed earlier in chicken primary myoblasts (on DM3) than in the *chTERT*-myoblasts at PDs 5 and 15 (on DM5) (Figure 4A). On the other hand, the immunofluorescence staining showed that under the same differentiation time (on DM5), the primary myoblasts formed significantly more myotubes compared with the *chTERT*-myoblasts at PDs 5 and 15 (Figure 4B). Subsequently, the temporal expression differences of the *MyHC* transcription levels from the growth medium (GM) to 5 days of DM were quantified in the primary myoblasts and *chTERT*-myoblasts, respectively. It was observed that the expression levels of *MyHC* in the primary myoblasts reached its peak earlier, while the *MyHC* expression in the *chTERT*-myoblasts at PDs 5 and 15 gradually peaked with differentiation (Figure 4C), indicating faster differentiation kinetics in the primary myoblasts compared with the *chTERT*-myoblasts. The Western blot results further demonstrated that despite undergoing the same differentiation time, the protein expression levels of the differentiation marker proteins (Myomaker and MyHC) were significantly higher in the primary myoblasts compared with the *chTERT*-myoblasts at PDs 5 (Figure 4D) and 15 (Figure 4E), suggesting that the *chTERT*-myoblasts maintained the capacity to differentiate but exhibited slower differentiation kinetics than the primary myoblasts.

### 2.5. Transfection Efficiency of chTERT-Myoblasts Was Sufficient

**Figure 5:**
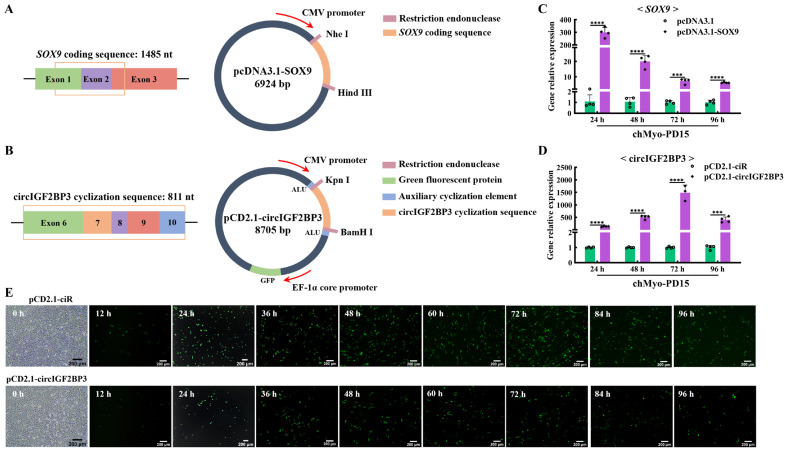
Analysis of transfection efficiency of *chTERT*-myoblasts. (**A**) Schematic diagram of pcDNA3.1-SOX9 structure. The orange box represents the coding sequence of *SOX9.* (**B**) Schematic diagram of pCD2.1-circIGF2BP3 structure. The orange box represents the cyclization sequence of circIGF2BP3. (**C**) Relative expression levels of *SOX9* in *chTERT*-myoblasts at PD 15 transfected with pcDNA3.1-SOX9 (mean ± SD; *** *p* < 0.001; **** *p* < 0.0001; *n* = 4). (**D**) Relative expression levels of *circIGF2BP3* in *chTERT*-myoblasts at PD 15 transfected with pCD2.1-circIGF2BP3 (mean ± SD; *** *p* < 0.001; **** *p* < 0.0001; *n* = 4). (**E**) Bright-field image before cell transfection and the fluorescence images of *chTERT*-myoblasts at PD 15 transfected with pCD2.1-circIGF2BP3.

Given that the chicken myoblast cell line we established was done so to facilitate the verification of candidate genes molecular functions in chicken myoblasts, we selected a gene overexpression vector and a circular RNA overexpression vector to conduct overexpression experiments by assessing the transfection efficiency for this cell line. To assess the transfection efficiency of the *chTERT*-myoblasts, the linear gene *SOX9* and the circular RNA circIGF2BP3 were selected for the overexpression validation. These genes were cloned into pcDNA3.1 and pCD2.1-ciR vectors, respectively (Figure 5A,B). The results showed that the *SOX9* overexpression significantly increased the expression of *SOX9* in the *chTERT*-myoblasts at PD 15 (Figure 5C). Similarly, the overexpression of circIGF2BP3 by pCD2.1-circIGF2BP3 led to a significant increase in the circIGF2BP3 expression in the *chTERT*-myoblasts at PD 15 (Figure 5D). Since the pCD2.1-circIGF2BP3 vector carried a green fluorescence protein tag, fluorescence microscopy results revealed the presence of green fluorescence proteins in the *chTERT*-myoblasts at PD 15 transfected with pCD2.1-circIGF2BP3 (Figure 5E). These results underscored that the transfection efficiency of our *chTERT*-myoblasts was sufficient to support the study of functional genes related to muscle development.

## 3. Discussion

In recent years, studies related to tumors showed that the activation of telomerase plays a crucial role in cell immortalization and is considered an indicator of cellular cancerization [29,30]. In contrast, normal somatic cells, which lack telomerase activity, undergo senescence and apoptosis with progressive telomere shortening after multiple population doublings [31]. It was demonstrated that the fine alternative splicing process after the *TERT* transcription, that is, the abundance of functional *TERT* mRNA, is one of the keys to regulating telomerase activity [32,33].

Currently, extensive and comprehensive research is being conducted and shows the potential of *hTERT* for cell immortality. For example, the activation of *hTERT* is regulated by multiple signaling pathways, such as Wnt/β-catenin, NF-κB, and c-myc, in different human tumors. By either solitary expression or in concert with oncogenes, *hTERT* enables a variety of human and other mammalian somatic cells to surpass replicative senescence and acquire immortality [34,35]. Furthermore, in the case of the chicken liver cancer cell line (LMH), it was observed that the chTERT protein can interact with the Wnt/β-catenin signaling pathway to inhibit cell apoptosis, promote cell proliferation and migration, and upregulate the expression level of the proto-oncogene *c-myc* [36]. Therefore, it is reasonable for us to utilize *chTERT* to restore the cellular telomerase activity and confer immortalization to chicken cells. Previous study corroborated the effectiveness of this approach, that is, ICP1 established by packaging the pLXRN-chTERT retrovirus and infecting chicken primary preadipocytes survived more than 100 population doublings [23]. Furthermore, the chicken skeletal muscle satellite cell line (ICMs) obtained by the same method also successfully bypassed replicative senescence and was cultured to population doubling 22 [37]. In this study, we successfully established a chicken myoblast cell line that retained the capacities for proliferation and partial differentiation by transduction of *chTERT* into primary myoblasts. Our result was consistent with the research that showed that the transduction of chTERT alone can restore chicken telomerase activity and immortalize chicken preadipocytes [23].

Previous research suggested that plasmid DNA can trigger cellular immune stimulation responses due to the relatively higher frequency of unmethylated CpG oligodeoxynucleotide sites in bacterial DNA compared with mammalian DNA. The innate immune system recognizes these CpG sites and removes plasmid DNA from the cytoplasm or nucleus [38]. As a result, a part of the cells loses the transfected nucleic acid molecules during cell division. Only a small portion of nucleic acid molecules manage to traverse the physical barrier of the cell membrane, entering the nucleus from the cytoplasm and integrates into the cell chromosomes through non-homologous recombination within the initial few hours after transfection [39]. It was speculated that the downregulation of *chTERT* expression after population doubling nine could be caused by part of the loss of the plasmid during a population doubling.

At present, there is a relative lack of understanding about the regulatory mechanism of myoblast fusion. It is widely recognized that myogenesis begins with the specific differentiation of precursor cells into myoblast lineage, which is mainly regulated through muscle-specific transcription factors, such as myogenic differentiation (MyoD) and myogenin (MyoG) [40]. In addition, myomaker (Mymk), as a multi-pass transmembrane protein, is also a muscle-specific cell fusogen [40]. In the current study, while the expression of *MyHC* in the *chTERT*-myoblasts at PDs 5 and 15 during differentiation did not reach its peak at a rapid rate, its expression was able to gradually and steadily increase as the differentiation progressed. This indicates that although the *chTERT*-myoblasts did not differentiate as fast as the primary myoblasts, they still maintained a certain differentiation capacity. Most of the skeletal myoblast cell lines obtained through different methods have the potential to differentiate into multinucleated muscle fibers [5,6,7,8], while there are also many myoblast cell lines have an extended lifespan and impaired myogenic differentiation potential [41,42]. It has been reported that six rat myoblast cell lines derived through exposure to methylcholanthrene (a carcinogen) retained the capacity for differentiation, yet were unable to form muscle fibers in a certain period of time [5]. Previous study has also reported that some mononuclear myoblasts isolated from the thigh muscles of dystrophic mouse cannot fuse into multinucleated muscle fibers [6]. The cells are arranged to form ”pseudo-straps”, which may be related to the presence of a large number of non-myogenic cells [6]. However, the myogenic cell purity of our *chTERT*-myoblasts was nearly 100%, and the *chTERT*-myoblasts at PDs 5 and 15 expressed a detectable amount of Mymk and MyHC, despite its protein expression levels for these proteins being lower than those observed in primary myoblasts under the same differentiation time. This further confirmed that while the differentiation kinetics of our *chTERT*-myoblasts were lower compared with the primary myoblasts, this cell line nonetheless consistently maintained a degree of myogenic differentiation capacity. Increasing evidence suggests that autophagy plays a crucial role in myogenic differentiation [43,44]. Moreover, iron overload has been reported to suppress the differentiation of C2C12 myoblast cells [45]. Therefore, it is worth further investigating whether the immortalization process led to impaired autophagy or iron overload in our cell line, which may have consequently affected the cell differentiation.

It has been reported that chromosomal abnormalities are common among cell lines transformed by SV40 T antigens, which may impact the generalizable nature of cell lines to species biology [46]. Viral oncogenes, such as SV40 Large-T antigen and adenoviruses E1A and E1B, were employed to immortalize avian cell lines. However, the main limitation of this approach is that the resulting cell lines frequently exhibit a loss of cell cycle and apoptosis control due to the inhibition of the pRB and p53 pathways, respectively, which ultimately leads to malignant transformation of the immortalized cells [47,48]. For the majority of human and other mammalian cell types, the human telomerase reverse transcriptase (*hTERT*) subunit represents the rate-limiting component of the telomerase enzyme complex [22,49]. The ectopic expression of *hTERT* alone can extend the cellular lifespan and immortalize a variety of cell types without inducing a malignantly transformed phenotype. In the current study, no viral oncogenes were introduced into the cell lines we established. This may partially account for the retention of differentiation capacity in our cell line without malignant transformation. In addition, as reported in prior studies, the transduction of the *chTERT* subunit alone was sufficient to immortalize the chicken preadipocytes. However, the combination of *chTERT* and *chTR* increased the telomerase enzymatic activity and facilitated more efficient and rapid cellular immortalization compared with the *chTERT* transduction alone [23]. Therefore, whether the combination of *chTERT* and *chTR* transduced into chicken myoblasts could achieve a better immortalization is worth further exploration.

The proliferation and differentiation of myoblasts are crucial for muscle development, which remains a continuing focus for the poultry industry. Therefore, the establishment of immortalized chicken myoblasts with preserving characteristics of proliferation and differentiation would be of great benefit to muscle development research. Our establishment of immortalized chicken myoblast cell lines provides a potential in vitro model to explore the molecular mechanisms underlying muscle development.

## 4. Materials and Methods

### 4.1. Ethics Statement

All animal experimental protocols were carried out in accordance with “The Instruc-tive Notions with Respect to Caring for Laboratory Animals” issued by the Ministry of Science and Technology of the People’s Republic of China and approved by the Institutional Review Board of Guangdong Ocean University (SYXK-2021-0154).

### 4.2. Isolation and Purification of Chicken Primary Myoblasts

Chicken primary myoblasts were collected from the thigh muscle tissue (approximately 1.75 g) of 10.5-day-old embryonic Yuexi frizzed feather chickens, which were purchased from Nanxia Village Chicken Farm in Mazhang District, Zhanjiang City, Guangdong, as previously documented [50]. Briefly, muscle tissue was isolated and meticulously minced into approximately 1 mm^2^ sections using scissors within an ultra-clean bench (Suzhou Antai Airtech Co., Ltd., Suzhou, China). Concurrently, the skin and bones were removed. After washing with phosphate buffered saline (PBS, Gibco, Grand Island, NY, USA), the muscle tissue was subjected to enzymatic digestion with 0.25% trypsin (Gibco, Grand Island, NY, USA) within a cell culture incubator set at 37 °C for 20 min, where approximately 3.5 mL of trypsin solution was used per g of muscle tissue. Subsequently, the digestion was terminated, and the undigested tissue underwent filtration successively through 100 mesh, 200 mesh, and 70 μm nylon screens. Eventually, the cells were collected by centrifugation at 395× *g* for 5 min at 37 °C and placed into a cell culture dish. The cell suspension was transferred to a new dish every 40 min, which was repeated twice to remove fibroblasts and epithelial cells. Finally, the cell suspension was transferred to a 100 mm cell culture dish (704001, NEST, Wuxi, China). About 48 h later, cells that almost covered the surface were collected and inoculated into plates for proliferation and differentiation analysis.

### 4.3. Cell Culture Conditions and Differentiation

Chicken primary myoblasts and DF-1 cells were cultured under a specific growth medium (GM), which contained 20% fetal bovine serum (FBS, PA500, NEWZERUM, Christchurch, New Zealand) + 1% penicillin/streptomycin (15140122, Gibco, Grand Island, NY, USA) + 79% RPMI 1640 medium (C11875500BT, Gibco, Grand Island, NY, USA), as well as 10% FBS + 1% penicillin/streptomycin + 89% DMEM medium (Gibco, Grand Island, NY, USA). Both cell types were cultured in an aseptic incubator set at 37 °C with 5% CO_2_ and passaged at a ratio of 1:2 when the cell confluence reached approximately 90%. During the differentiation of myoblasts, the medium containing 20% FBS was substituted with a medium containing 2% horse serum (HS, 26050088, Gibco, Grand Island, NY, USA), 100 units/mL penicillin, and 100 µg/mL streptomycin (final concentration, Gibco, Bethesda, MD, USA), which was referred to as the differentiation medium (DM). The relative myotube area (%) referred to the ratio of the number of nuclei contained in MyHC positive cells to the total number of nuclei in a certain field of view.

### 4.4. Determining the Optimal Antibiotic Screening Concentration

In the quest to identify the optimal antibiotic screening concentration, the following procedure was employed: chicken primary myoblasts were collected and cultured in 24-well plates (702001, NEST, Wuxi, China), with about 1 × 10^5^ cells per well. After 24 h of adhesion, the cell confluence in each well reached approximately 70%, and the culture medium was replaced with a selective medium that contained different concentrations of Geneticin (G418). Antibiotic solution with a concentration of 50 mg/mL was prepared using sterilized double-distilled water (ddH_2_O) and G418 powder (AG138-1G, Genview, Beijing, China). The prepared solution was then filtered through 0.22 μm sterile needles (SLGPR33RB, Merck KGaA, Darmstadt, Germany) and stored at −20 °C. For the chicken primary myoblasts, various concentration gradients were used: 0, 25, 50, 75, 100, 150, 200, and 400 μg/mL. Four replicates were established for each concentration. Fresh selection medium corresponding to the designated concentration was replaced every three days for 10 to 14 d.

### 4.5. Screening of the Chicken Myoblast Cell Line with chTERT Overexpression

A total of 2 × 10^5^ chicken primary myoblasts were collected and seeded into separate wells of 12-well plates (712001, NEST, Wuxi, China). Recombinant plasmid pLXRN-chTERT was obtained from Professor Li’s research group of Northeast Agricultural University. When the cell confluence reached about 80%, the pLXRN-chTERT overexpression vector was introduced into the cells using lipo8000^TM^ transfection reagent (C0533, Beyotime, Shanghai, China), and a control group without any treatment. After 48 h, the culture medium was replaced with an optimal concentration of G418 selection medium. Following one week of continuous screening, the concentration of G418 in the selection medium was halved. The selection process continued until all cells in the control group without treatment had died. At this point, the 1st generation of the myoblasts that stably expressed *chTERT* (*chTERT*-myoblasts) was successfully obtained. Finally, the myogenic properties of the *chTERT*-myoblasts were assessed. Additionally, the expression of the *chTERT* gene at the mRNA level was examined.

### 4.6. RNA Isolation and cDNA Synthesis

Total RNA was extracted from cell samples at different passages and time points according to the instructions provided by the HiPure Universal RNA Kit (R4130-03, Magen Biotechnology Co., Ltd., Guangzhou, China). The concentrations of the RNA samples were measured and recorded using a NanoDrop TM Lite spectrophotometer (Thermo Fisher Scientific, Waltham, MA, USA). Subsequently, the first-strand cDNA was synthesized from 1 μg of RNA template with a primer mix (including oligo dT and random primers) using HiScript III RT SuperMix for qRT-PCR (+gDNA wiper) (R323-01, Vazyme Biotech Co., Ltd., Nanjing, China). The reaction system and procedure are shown in Table S1.

### 4.7. Quantitative Real-Time PCR (qRT-PCR)

All qRT-PCR experiments were conducted with ChamQ Universal SYBR qPCR Master Mix (Q711-02, Vazyme Biotech Co., Ltd., Nanjing, China) on the CFX Connect Real-Time System (BIO-RAD, Singapore). Each experiment was carried out with three replicates. The relative expression levels of mRNA were quantified using the comparative 2^−ΔΔCt^ method. The forward and reverse primers for *chTERT* and *GAPDH* (used as an internal control gene) were designed with the primer-BLAST tool (https://blast.ncbi.nlm.nih.gov/Blast.cgi; accessed on 18 July 2022). All primer sequences are shown in Table S2. The qRT-PCR reaction proceeded as follows: pre-denaturation at 95 °C for 30 s; denaturation at 95 °C for 5 s; and annealing at 60 °C and extension for 30 s, cycling 40 times. The reaction system was shown in Table S3.

### 4.8. Western Blot

The detailed procedures for total protein extraction and Western blot followed the previous report by López et al. [7]. RIPA Lysis Buffer (P0013B, Beyotime, Shanghai, China) and PMSF Solution (100 mM, ST507, Beyotime, Shanghai, China) were used. The antibodies used for the Western blot analysis included anti-Desmin, anti-MyoD1, anti-GAPDH, anti-Cyclin D1 (1:500 dilution, Abmart, Shanghai, China), anti-Mymk (1:500 dilution, Abclonal, Wuhan, China), anti-MyHC (1:500 dilution, DHSB, Iowa, IA, USA), and HRP-labeled goat anti-rabbit IgG (1:500 dilution, Beyotime, Shanghai, China), which were diluted by a primary antibody dilution buffer (P0023A, Beyotime, Shanghai, China) and secondary antibody dilution buffer (P0023D, Beyotime, Shanghai, China). In addition, the SDS-page gel and blotting equipment involved in the experiment included a Mini-PROTEAN^®^ Tetra Cell Casting Module (1658015, BIO-RAD, Singapore) and Mini-PROTEAN^®^ Tetra Vertical Electrophoresis Cell for Mini Precast Gels, 4-gel, Mini Trans-Blot^®^ Module, and PowerPac^TM^ HC Power Supply (1658036, BIO-RAD, Singapore). Before incubating the primary antibodies, the PVDF membranes (IPVH00010, Merck Millipore Ltd., Darmstadt, Germany) that contained the protein were immersed in QuickBlock^TM^ blocking buffer (P0252, Beyotime, Shanghai, China) for 1 h.

### 4.9. Immunofluorescence Assay (IFA)

Cultured cells and myotubes in 12-well plates (712001, NEST, Wuxi, China) were washed with PBS and fixed with 4% pre-cooled paraformaldehyde (P0099, Beyotime, Shanghai, China) at room temperature for 20 min. After fixation, the cells were permeabilized with 0.1% Triton X-100 (P0096, Beyotime, Shanghai, China) and then incubated with a blocking solution (E674004-0100, Shanghai Sangon Biotech Co., Ltd., Shanghai, China) to prevent non-specific binding. The rabbit primary antibody Desmin (1:100 dilution, PA1337S, Abmart, Shanghai, China) was added to the cells and allowed to incubate overnight at 4 °C. Subsequently, the cells were exposed to FITC-labeled goat anti-rabbit IgG (1:100 dilution, A0562, Beyotime, Shanghai, China) and incubated in the dark for 2 h. The cell nuclei were stained with DAPI at a concentration of 5 mg/mL (E607303-0020, Shanghai Sangon Biotech Co., Ltd.), and the stained cells were observed under a fluorescence microscope (OLYMPUS, Tokyo, Japan) using Image J software v1.53. After each step, the cells needed to be washed with PBS to remove excess reagents and ensure accurate staining. Furthermore, after the differentiation induced by 2% HS, the rabbit primary antibody MyHC (1:100 dilution, DHSB, Iowa, IA, USA) and Cy3-labeled goat anti-rabbit IgG (1:100 dilution, A0516, Beyotime, Shanghai, China) were applied following a similar staining procedure.

### 4.10. Cell Counting Kit-8 (CCK-8) Assay

A total of 3 × 10^3^ myoblasts were collected and seeded into separate wells of a 96-well plate (701001, NEST, Wuxi, China). At 0, 24, 48, and 72 h after the cell adhesion, the cells were incubated in the GM containing 10% CCK-8 reagent (GK10001, GLPBIO, Montclair, CA, USA) for 1 h. After that, the absorbance of each well was measured at 450 nm using a microplate reader (Beijing Perlong New Technology Co., Ltd., Beijing, China), and the collected data were used to construct a cell proliferation curve.

### 4.11. 5-Ethynyl-2′-Deoxyuridine (EdU) Assay

After 2 h of culture with the EdU reagent, the primary myoblasts and *chTERT*-myoblasts (adherent growth) in a logarithmic growth phase were fixed, permeabilized, and stained according to the instructions provided in the EdU Apollo In Vitro Imaging Kit (C10310-1, RiboBio, Guangzhou, China). Subsequently, four random regions were selected and the number of stained cells were assessed using fluorescence microscopy (IXplore standard, OLYMPUS, Tokyo, Japan).

### 4.12. Cell Counting

The primary myoblasts and *chTERT*-myoblasts with 1 × 10^5^ cells were inoculated into separate 24-well plates (702001, NEST, Wuxi, China). Over the culturation of one week, the number of living cells in each well was calculated every 24 h using a blood cell counter in conjunction with trypan blue staining. The cells were digested with 300 μL trypsin to detach from the surface. Three replicates were maintained for each group. Based on the daily cell counts, a growth curve was drawn.

### 4.13. Serum Dependence Analysis

The cell viability under different concentrations of serum culture was assessed using the CCK-8 assay. The *chTERT*-myoblasts were collected and seeded in a 96-well plate (701001, NEST, Wuxi, China) with 8 × 10^3^ in each well. After 24 h of culture, the 20% FBS RPMI 1640 medium was replaced with medium containing varying concentrations of FBS, including 0, 5, 10, and 20%. Cells were continuously cultured for 72 h under these different serum concentration conditions. The optical density at 450 nm (OD450) values were detected every 24 h using the CCK-8 assay. Six replicates were maintained for each serum concentration.

### 4.14. Statistical Analysis

The data were presented as the mean value ± standard error and plotted using GraphPad Prism 9.0.0 (GraphPad Software, Boston, MA, USA) to create graphical representations. The statistical analyses were performed using the two-tailed Student’s *t*-test for comparing two groups, or the one-way analysis of variance with Tukey’s multiple comparison test for comparing between multiple groups. A significance level of *p* < 0.05 was considered statistically significant.

## 5. Conclusions

**Figure 6:**
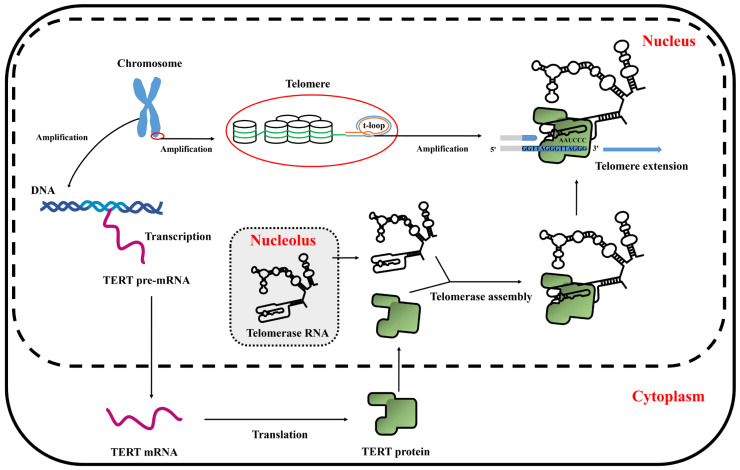
The schematic diagram of TERT promoting telomere elongation.

In this study, we successfully established a chicken myoblast cell line, which was designated as *chTERT*-myoblasts, for the first time. These cells exhibited remarkable ability to maintain the morphology, proliferation, differentiation, and transfection capacity akin to that of primary myoblasts. However, the proliferation rate of *chTERT*-myoblasts with different population doublings differed from the primary cells, and it exhibited slower differentiation kinetics. A schematic diagram of TERT promoting telomere elongation was shown in Figure 6.

## Data Availability

All data generated or analyzed during this study are included in this published article and Supplementary Materials.

## Supplementary Materials

The following supporting information can be downloaded from https://www.mdpi.com/article/10.3390/ijms25158340/s1.
