# Supplementary material for: Establishment and Characterization of a Chicken Myoblast Cell Line
Source: Int J Mol Sci. 2024 Jul 30;25(15):8340. doi: 10.3390/ijms25158340 (PMC11312951; doi:10.3390/ijms25158340)
Supplement: Supplementary file 1 [file ijms-25-08340-s001.zip › ijms-3064875-supplementary.pdf]

Table S1. Reverse transcription reaction system and procedure.

	Reagent Name	Use Level
Step 1 [1]	4 × gDNA wiper Mix	4 μL
	Template RNA	1 μg
	RNase-free ddH_2O	to 16 μL
Step 2 [2]	Step 1 reaction liquid	16 μL
	5 × HiScript III qRT SuperMix	4 μL

[1] Reaction at 42°C for 2 min. [2] 37°C for 15 min, 85°C for 5 s, 4°C forever.

Table S2. Primers used for expression analysis.

Primer Name	Forward Primer (5'-3')	Reverse Primer (5'-3')	Annealing Temperature (°C)	Product Length (bp)
chTERT	CAAGGCTCCGGTTCATTCCT	TCCCGAATACTGAAGAGCCA	60	201
MyoD1	GCTACTACACGGAATCACCAAAT	CTGGGCTCCACTGTCACTCA	60	200
MyoG	CGGAGGCTGAAGAAGGTGAA	CGGTCCTCTGCCTGGTCAT	60	320
Desmin	GCCAGCGAAGGAATGAGC	CGTCCGATTGGATAGACAGAAC	60	138
MKI67	ATTCGCATCCACTTGCCTCA	TGCTGAACATGAAGAACCTGC	60	269
c-myc	GCCAGCGAAGGAATGAGC	CGTCCGATTGGATAGACAGAAC	60	138
CDKN1A	GCAGCAAAGCGTGCAGGAA	GCGTCTCGGTCTCGAAGTTGA	60	132
MyHC	CTCCTCACGCTTTGGTAA	TGATAGTCGTATGGGTTGGT	60	213
Mymk	TGGGTGTCCCTGATGGC	CCCGATGGGTCCTGAGTAG	60	135
Cyclin D1	AACCCACCTTCCATGATCGC	CTGTTCTTGGCAGGCTCGTA	60	159
circIGF2BP3	TCTGAATGCCTTGGGTCTGTT	GTCCAAATCCACGTCGTCCC	64.5	228
SOX9	GACAAATGCATCTCCGACGC	GCTTCACGTGGGGTTTGTTC	60	277
GAPDH	AGGACCAGGTTGTCTCCTGT	CCATCAAGTCCACAACACGG	60	153

Table S3. qRT-PCR reaction system.

Reagent Name	Use Level (μL)
Template cDNA [1]	2
Forward primer	0.4
Reverse primer	0.4
2 × ChamQ Universal SYBR qPCR Master Mix	10
RNase free water	7.2

[1] Dilution quadruple.
